# Accuracy and limitations of vector flow mapping: left ventricular phantom validation using stereo particle image velocimetory

**DOI:** 10.1007/s12574-016-0321-5

**Published:** 2016-11-15

**Authors:** Rei Asami, Tomohiko Tanaka, Ken-ichi Kawabata, Kunio Hashiba, Takashi Okada, Tomohide Nishiyama

**Affiliations:** Hitachi Ltd, 1-280 Higashi-Koigakubo, Kokubunji, Tokyo Japan

**Keywords:** Doppler ultrasound, Ultrasonics, Flow imaging, Cardio-hemodynamics

## Abstract

**Background:**

The accuracy of vector flow mapping (VFM) was investigated in comparison to stereo particle image velocimetry (stereo-PIV) measurements using a left ventricular phantom. VFM is an echocardiographic approach to visualizing two-dimensional flow dynamics by estimating the azimuthal component of flow from the mass-conservation equation. VFM provides means of visualizing cardiac flow, but there has not been a study that compared the flow estimated by VFM to the flow data acquired by other methods.

**Methods:**

A reproducible three-dimensional cardiac blood flow was created in an optically and acoustically transparent left-ventricle phantom, that allowed color-flow mapping (CFM) data and stereo-PIV to be simultaneously acquired on the same plane. A VFM algorithm was applied to the CFM data, and the resulting VFM estimation and stereo-PIV data were compared to evaluate the accuracy of VFM.

**Results:**

The velocity fields acquired by VFM and stereo-PIV were in excellent agreement in terms of the principle flow features and time-course transitions of the main vortex characteristics, i.e., the overall correlation of VFM and PIV vectors was *R* = 0.87 (*p* < 0.0001). The accuracy of VFM was suggested to be influenced by both CFM signal resolution and the three-dimensional flow, which violated the algorithm’s assumption of planar flow. Statistical analysis of the vectors revealed a standard deviation of discrepancy averaging at 4.5% over the CFM velocity range for one cardiac cycle, and that value fluctuated up to 10% depending on the phase of the cardiac cycle.

**Conclusions:**

VFM provided fairly accurate two-dimensional-flow information on cardio-hemodynamics. These findings on VFM accuracy provide the basis for VFM-based diagnosis.

## Introduction

An understanding of left ventricle (LV) flow dynamics, which is known to be multidirectional, asymmetrical, and vortical [1], will enable diagnosis of cardiac abnormalities. For example, vortex formation and recirculation patterns in the LV have been reported to differ in cases of dilated cardio-myopathy [2]. Several reliable methods of visualizing intracardiac flows [3], such as cardiac magnetic resonance (CMR), echocardiography particle image velocimetry (echo-PIV), and vector-flow mapping (VFM), have been developed. Of these methods, VFM can easily and non-invasively visualize 2D blood flows [4] as the method is based on conventional ultrasound scanner and does not require administration of contrast agents.

**Fig. 1:**
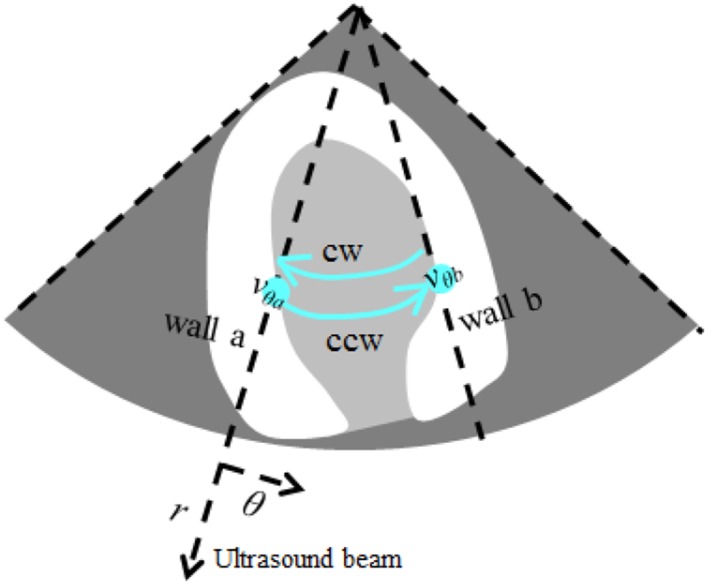
Schematic of VFM algorithm

VFM data is obtained on the basis of blood-flow velocity in the LV measured by color-flow mapping (CFM) and cardiac wall velocity acquired by tissue tracking. The mass-conservation law is applied to CFM velocity to estimate the azimuth velocities under the assumption that the flow is 2D [5–7]. Original approach [5, 8] calculates the stream function, which is the integral form of the 2D continuity equation. Recent methods [6, 7] directly solve the 2D continuity equation, and use cardiac wall velocity acquired by tissue tracking in order to improve the algorithm (Fig. 1) (see “Appendix 1” for derivation of VFM). The simplicity of the VFM methodology makes it a potent diagnostic tool in that it only requires readily available CFM and does not require a contrast agent.

To fully extend the use of VFM in clinical scenarios, the accuracy and limitations of VFM when applied to cardiac flows must be investigated. The VFM algorithm was numerically validated by using symmetrical and two-dimensional flow fields [8]. An algorithm to estimate the flow field similar to that of VFM has been validated using a symmetrical heart phantom [6], which is expected to satisfy the 2D flow assumption. This study also compared the algorithm with CFM measurements and found that violation of the 2D-flow assumption accounted for close to 15% error, suggesting the importance of direct validation of VFM in a 3D flow field.

The main aim of this study was to qualitatively and quantitatively evaluate the accuracy of VFM in a 3D LV blood flow field by comparing VFM with established flow measurements, namely, those from stereo particle image velocimetry (stereo-PIV or PIV). An in-house LV phantom mimicking the anatomy of a healthy individual was developed and used to create a reproducible three-dimensional flow. As the phantom could optically and acoustically be measured, VFM data could be compared with stereo-PIV data, and VFM accuracy could be evaluated.

## Materials and methods

### Overview of experimental setup

**Fig. 2:**
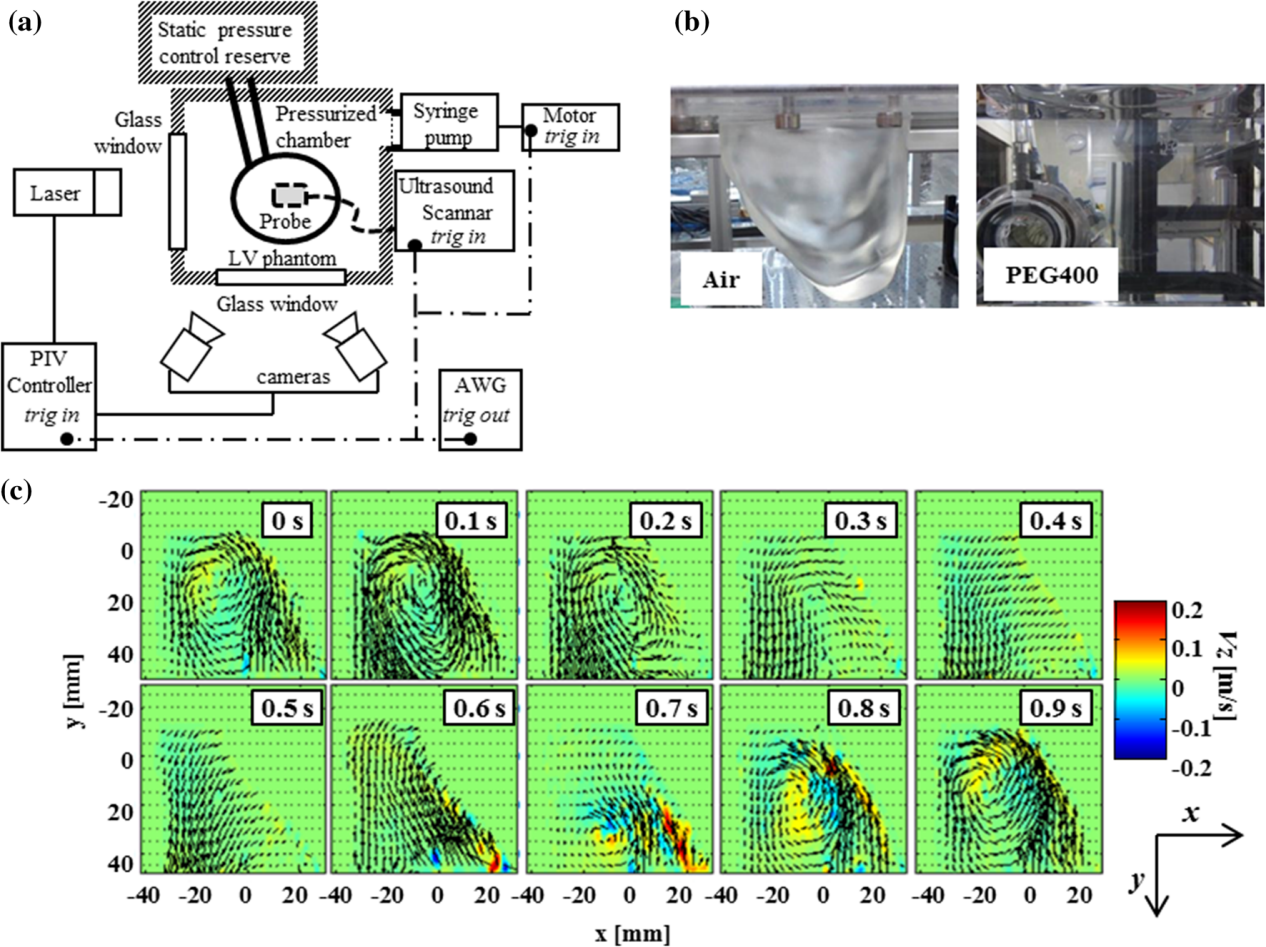
Experimental system: **a** Schematics of the experimental setup, which consists of an LV phantom, an ultrasound scanner, and a PIV. A pulse signal mimicking an R-wave generated by an activator synchronizes all three systems at 1 Hz. **b** LV phantom in air (*left*) and in PEG400 (*right*) and **c** stereo-PIV velocity mapping of LV phantom. *Contour* indicates through-plane velocity component *v*_z_. *Time above* each *frame* indicates duration after triggering pulse

Validity of VFM was experimentally investigated by using an in-house-developed pulsatile LV phantom. A top view of the experimental facility, which consists of an LV phantom, an ultrasound scanner, and a stereo-PIV system, is shown in Fig. 2a. A pulse generator (33220A, Agilent Technologies, Inc., USA) activated these instruments at 1 Hz to acquire synchronized data sets.

### Left-ventricular phantom

An LV phantom that was made from transparent soft urethane resin (Exseal Corporation, Japan) was molded on the basis of human-LV computer-aided-design (CAD) data (model No. 2, Virtual Anatomia, Japan SGI, Japan). The molded phantom was enlarged by a factor of 1.6 and was translucent in air (left of Fig. 2b). The measured refractive index of the phantom was 1.47 at a light wavelength (λ) of 532 nm. Young’s modulus of the soft urethane resin was measured to be approximately 57 kPa (at 1% strain) by using a soft-tissue elastometer [9]. Two bileafelet mechanical valves (the first with a diameter of 25 and the second with a diameter of 28 mm) were inserted into the mitral (the first) and aortic (the second) positions. The refractive indices of the intracirculatory fluid and external fluid had to be matched with the refractive index of the LV phantom to eliminate any potential optical distortions. Polyethylene glycol (PEG) 400 (Wako Pure Chemical Industries, Ltd., Japan) with a refractive index of 1.47 at λ = 532 nm was selected for this purpose. The matched refractive indices made the LV phantom almost invisible (right of Fig. 2b). It was also important to ensure that the flow dynamics in the phantom were comparable to those of the actual hemodynamics. The Reynolds number of the experimental system was assumed to match that of an actual heart. This assumption was based on the fact that the viscosity of PEG 400 is approximately 1.5 times greater than that of blood, which compensates for the phantom size being 1.6 times greater than that of an actual heart. Tracer particles (Expancel 80, Japan Fillitte Co., Japan) were mixed with the intracirculatory fluid for tracking purposes to acquire PIV.

The LV phantom was fixed to an acrylic pressurized chamber with its valves facing upward. Tubes from a static-pressure control reserve were connected to the valves of the phantom to serve as an inlet and outlet for the intracirculatory fluid. A periodic and pulsatile flow was generated with an in-house syringe pump driven with a motor (F14-10, Yamaha Motor Co., Ltd., Japan) connected to the pressurized chamber. The LV phantom contracted and expanded by changing the pressure in the chamber, thereby producing outflow, inflow, and intracardiac flow. The pump created a volumetric change of ~75 mL, which resulted in an ejection fraction in the LV phantom of ~50%.

### Ultrasound measurements

An ultrasound scanner (ProSound^®^ α10, Hitachi Aloka Medical, Ltd., Japan) with a sector probe (UST-52105, Hitachi Aloka Medical Ltd., Japan) acquired color Doppler and B-mode images of about 15 heartbeats at a center frequency of 2.5 MHz. VFMs were calculated from acquired B-mode images and CFM data off-line with the VFM algorithm described in the previous section, which was incorporated into the data analysis system software (DAS-RS1, Hitachi Aloka Medical, Ltd., Japan). The tissue velocity of the cardiac wall used in the VFM algorithm was calculated as follows. Firstly, the cardiac wall was manually traced in a systolic frame. A moving-average filter was then applied to the datasets, before the pyramidal Kanade-Lucas Tomasi (KLT) tracker method [10] was applied to track the cardiac wall for all frames. Tissue velocities were calculated, and neighboring vectors were averaged. CFM data were preliminarily filtered in the depth and radial directions by using an averaging filter before the VFM was calculated.

Due to the difference in the speeds of sound in tissue and PEG400, the resultant VFM velocities, *v* ^eqp^, calculated by the ultrasound scanner were modified by simply multiplying the resulted vectors by a correction factor, *C*_*f*_, as follows:

1 $$ \overrightarrow {v} = C_{f} \overrightarrow {{v^{\text{eqp}} }} $$

2 $$ C_{f} = \frac{{c_{P} }}{{c_{b} }} $$

where *C*_*b*_ and *C*_*P*_ are the speeds of sound for bodies (i.e., 1530 m/s) and for PEG 400 (1610 m/s), respectively. The justification for the correction of the speed of sound is briefly described in “Appendix 2”.

### Stereo-particle image velocimetry

The Stereo-PIV acquired 3D velocity vectors components in 2D planer cross sectional fields of the phantom. Tracer particles (Expancel^®^ 80, Japan Fillite Co., Ltd., Japan) were mixed with the intracirculatory fluid for tracking purposes to acquire PIV. A Raypower 5000-PIV Nd:YAG laser (Dantec Dynamics, A/S, Denmark, with power of continuous 5 W at 532 nm and thickness of about 4 mm) illuminated the tracers in a cross-sectional plane containing both the valves and the apex at the middle of the phantom. Two adjacent cameras (SpeedSense1010, Dantec Dynamics, A/S, Denmark) with 50-mm micro-Nikkor lenses captured the tracer images at a frame rate of 250 Hz. To cancel out background noise, only tracer images were extracted by subtracting the background images. PIV vectors were calculated using commercial software (Dynamic Studio, Dantec Dynamics, A/S, Denmark). A standard cross-correlation algorithm with three-point Gaussian fitting [11] was used. The vector spacing was set to about 0.4 × 0.7 mm by using 8 × 32 pixel-interrogation windows with 50 and 75% overlaps. The PIV velocity maps generated by the two cameras were then merged to create a stereographic image containing through-plane velocity information. To evaluate the degradation of VFM accuracy, the VFM velocities were compared with those obtained from the PIV, which provides accurate 2D velocity components in a plane. The uncertainty of the three-point Gaussian fitting is typically expected to be about 0.2 pixels. To compare the VFM and PIV vectors, the same calibration board was used to unify the coordinate system. Both spatial and temporal resolutions matched. Since the obtained VFM spatial resolution was higher than the PIV resolution, the VFM vectors were spatially averaged in accordance with PIV grid size. On the other hand, eight frames of the PIV results in the same phase were averaged to increase the accuracy of the PIV vectors.

### Quantification of VFM accuracy

VFM velocity fields were compared with those by PIV. Correlations between the two velocity fields in a time-course manner were calculated. Probability density functions (PDF) and SD of the velocity errors defined as differences between PIV and VFM velocities were examined. Error [%] is defined as:

3 $$ {\text{Error}} = \frac{{\left| {v^{\text{VFM}} - v^{\text{PIV}} } \right|}}{{v^{\text{Range}} }} $$

where *v* ^VFM^ − *v* ^PIV^ and *v* ^Range^ are a discrepancy between velocity vectors and a full CFM velocity range, respectively.

### Vortex trajectory

Vortex trajectory was examined by visualizing flow circulation, which represents the angular momentum of flow. The circulation was calculated to roughly compare the outline of flow of VFM data to that of PIV data by tracing the vortices in a time-course manner. More precisely, to calculate map of circulation, square line integral of the 15 mm size in clockwise direction was applied. The local extremum of the magnitude of circulation, LEC, was interpreted as being where the flux of the vortices was at maximum, which is similar to the center of the vortices.

### Wall-motion analysis

The errors due to acoustic tissue tracking were evaluated since the VFM algorithm uses wall-velocity measurements obtained by tissue tracking. Wall-motion data were calculated from the PIV results for comparative purposes. The internal surfaces of walls were manually traced on a diastolic frame, and a fast method of cross-correlation [12] to register images was used to track wall displacement. Vectors outside the wall were excluded prior to any statistical analyses of PIV and VFM.

### PIV reconstruction for validation of planar flow

The VFM algorithm was applied to the PIV data to evaluate what influence the planar-flow assumption had on error. The vertical velocity, *v*_*y*_, of the PIV vectors was used instead of CFM velocity, and the wall velocity determined from wall-motion analysis was used instead of tissue-tracking data to calculate the *x*-directional, horizontal velocity, *v*_*x*_. All rows of the PIV vectors were calculated from left to right and vice versa, and a linear weighed function was applied to both sets of velocities to result in one set of horizontal velocities. Note that all calculations of PIV reconstruction were done with Cartesian coordinates instead of cylindrical coordinates. The calculated velocities in the Cartesian coordinates were converted to those in the cylindrical coordinates so that only the azimuthal velocities could be evaluated.

The violation of the planar-flow assumption was evaluated to further evaluate the 3D flow field by mapping *G*, defined by the following mass-conservation equation

4 $$ G = \frac{{\partial v_{x} }}{\partial x} + \frac{{\partial v_{y} }}{\partial y} $$

Under the assumption of planar flow, the value of *G* should be zero. Deviation of *G* from zero indicates the assumption has been violated.

## Results

3D velocity mapping of the LV phantom acquired by stereo-PIV for a single cardiac cycle is shown in Fig. 2c. Diastolic onset occurs at around 0.6 s, where rapid inflow begins. The late diastolic phase continues until 1.0 s (back to 0 s), after which the onset of the systolic phase begins. An asymmetrical through-plane flow is observed throughout the cardiac cycle. The fraction of the through-plane component, *v*_*z*_, over the magnitude of vector, |*v*|, was calculated to be 34% on average, with a maximal value of 44% observed at 0.66 s.

**Fig. 3:**
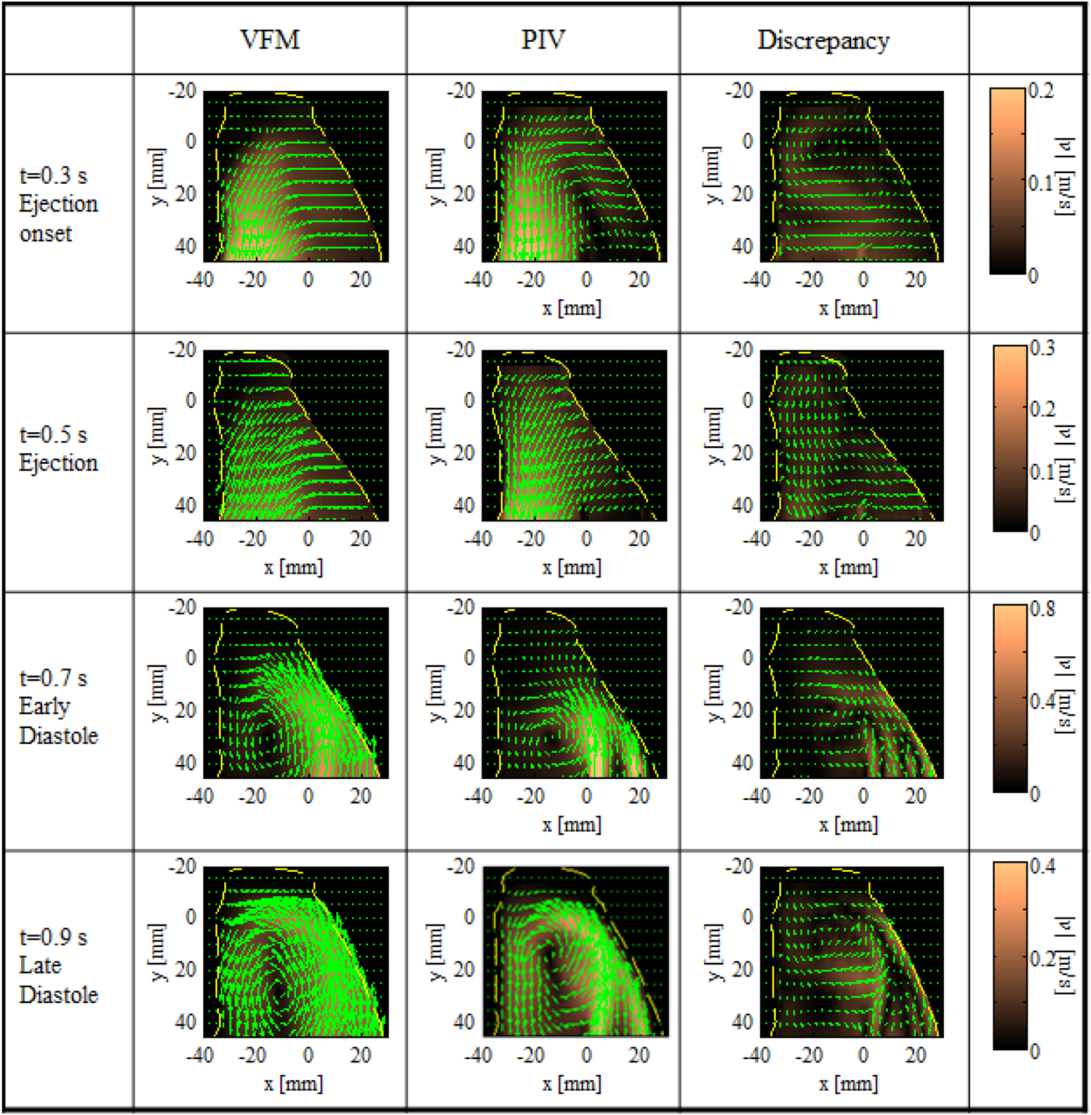
Comparison of 2D velocity fields. PIV, VFM, and their discrepancies (i.e., differences between PIV and VFM data) are recorded at different time frames. *Background color* indicates magnitude of 2D vectors. *Dashed lines* indicate wall boundaries acquired by PIV. *Color scale*, indicating magnitude of vector, is optimized for each time frame

The 2D velocity mapping acquired by PIV was compared with that acquired by VFM in Fig. 3. Two separate transmitral jets can be observed because of the bileaflet structure of the LV valves at 0.6 s. The flow then moves up along the left wall, while forming a vortex, and keeps moving toward the apex at 0.7 s. Contraction of the left wall occurs during the ejection phase, which causes the flow to exit from the aortic valves at the bottom left of each velocity mapping.

**Fig. 4:**
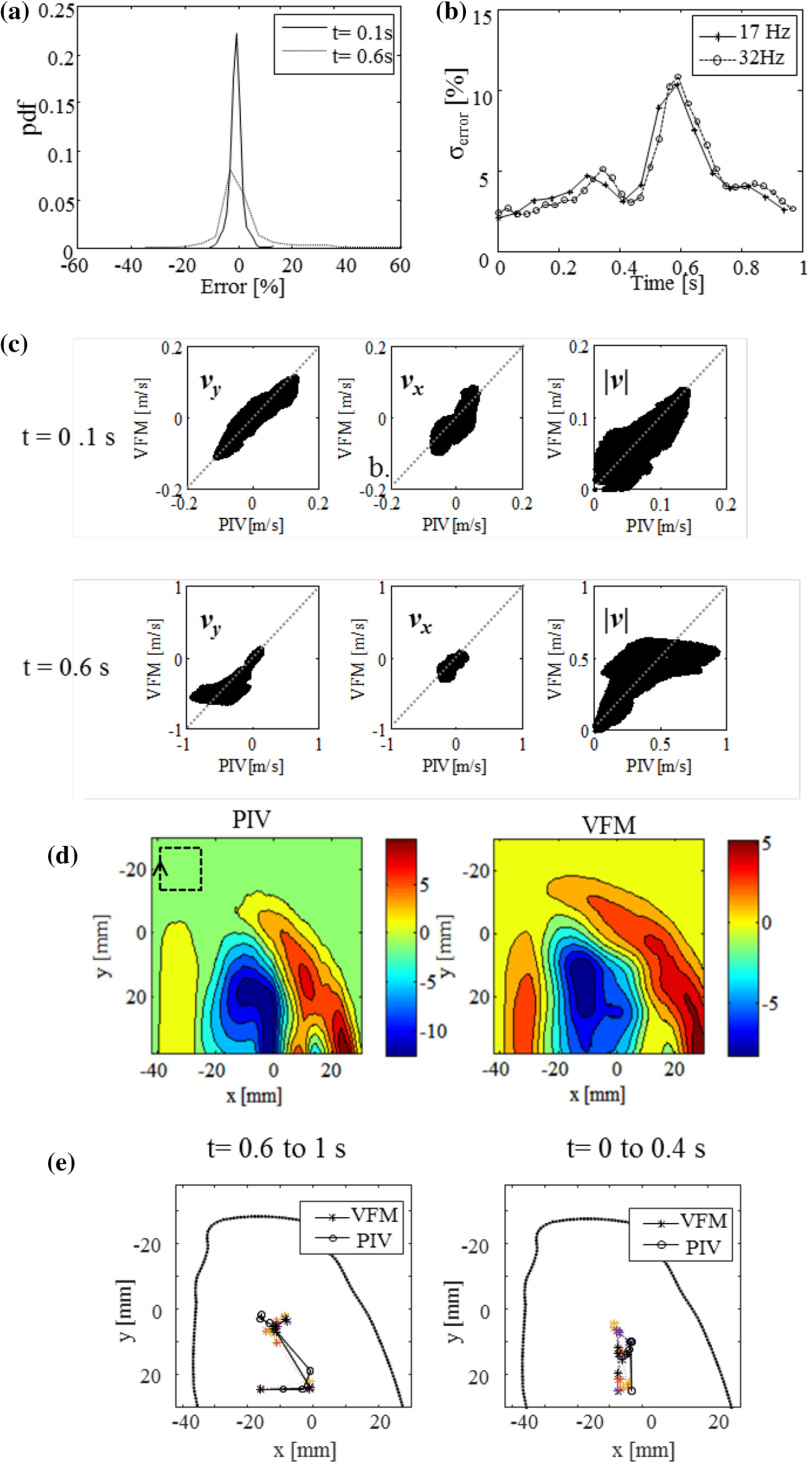
Statistical analysis: **a** Probability distribution function (pdf) of absolute velocity error, **b** transition in standard deviation of vector discrepancy over CFM velocity range, and **c** correlation between VFM and PIV vectors. All vector components are plotted in graphs at *t* = 0.1 s and 0.6 s for all frames. Velocity range is set to fit all *plots* for *both time frames*. *Dotted lines* indicate *y* = *x*. **d** Circulation mapped at *t* = 0.6 s. *Square* with *arrowhead* indicates size and direction of line integral applied to calculate maps of circulation. **e** Local extremum of circulation (LEC) has been *plotted* for time period to indicate transition pathway. VFM estimation data represents the average of three cycles. Each LEC for three cardiac cycles is mapped by *colored dotted line* to demonstrate reproducibility

The statistical distribution of velocity discrepancies, defined below, is plotted as a probability distribution function (pdf) of error in Fig. 4a.

Moderate circulating flow is observed at a peak speed of 0.14 m/s at 0.1 s, whereas rapid inflow is observed at a peak flow speed of 1 m/s at 0.6 s. The distribution peaks at 0.1 s at 0% with a mean value of 1% and a standard deviation (SD) of 3.8%. The distribution peaks slightly below zero at 0.6 s with a mean value of 0.5% and an SD of 10.5%. As seen in Fig. 4b, the SD for error is maximum at 0.6 s regardless of the frame rate. A minor peak, which corresponds to the peak ejection phase, can be observed around 0.3 s. The mean value for the SD of error in one whole cardiac cycle is 4.5%.

The correlation between the vectors of the VFM and PIV data is shown in Fig. 4c. Vertical component *v*_*y*_, horizontal component *v*_*x*_, and total magnitude |*v*| of the vectors are plotted separately. Each graph is fitted to a linear function by unconstrained nonlinear minimization of the sum of squared residuals. Calculated slope *a* and correlation coefficient *R* are summarized in Table 1. All correlations are statistically significant (*p* < 0.0001). High values of *R* for |*v*| suggest overall high levels of correlation between all vectors of VFM and PIV data. The slopes of all fitted curves are less than one, suggesting the velocities have been underestimated by VFM. The distribution appears to consist of two distinguishable groups at *t* = 0.6 s, in which one group exhibits a slope larger than one, which can be observed where the PIV velocity is under 0.3 m/s, and the other group exhibits a slope smaller than one observed where the PIV velocities are greater than 0.3 m/s.

**Table 1:** Summary of correlation parameters

	*t* = 0.1 s			*t* = 0.6 s			All		
	*v*_*y*_	*v*_*x*_	*|v|*	*v*_*y*_	*v*_*x*_	*|v|*	*v*_*y*_	*v*_*x*_	*|v|*
*A*	0.91	0.73	0.92	0.81	0.80	0.89	0.83	0.79	0.91
*R*	0.95	0.69	0.95	0.87	0.75	0.90	0.90	0.71	0.87

Flow circulation to represent the angular momentum of flow was calculated to roughly compare the outline of flow of VFM data to that of PIV data by tracing the vortices in a time-course manner (Fig. 4d). The local extremum of the magnitude of circulation, LEC, was interpreted as being where the flux of the vortices was at maximum, which is similar to the center of the vortices. The LECs have been plotted from 0.6 s to 1 s (rapid-inflow phase) and 0 s to 0.4 s (relatively slow flow phase) in Fig. 4e. Similar LEC transition patterns were observed both in the PIV and VFM results. Swirling motion forms in the left of the mitral valve, moves toward the center of the LV phantom, and stays there for both PIV and VFM during the rapid inflow phase. LEC, which was not observed for VFM, was observed in the transitional state from around the bottom to the center of the phantom for PIV. The LEC of VFM and that of PIV were the farthest apart at this instant, namely, 18 mm. LEC stayed around the center and moved toward the base of the phantom during the slow flow phase. The LECs of VFM and PIV were 4 mm apart at the farthest point.

**Fig. 5:**
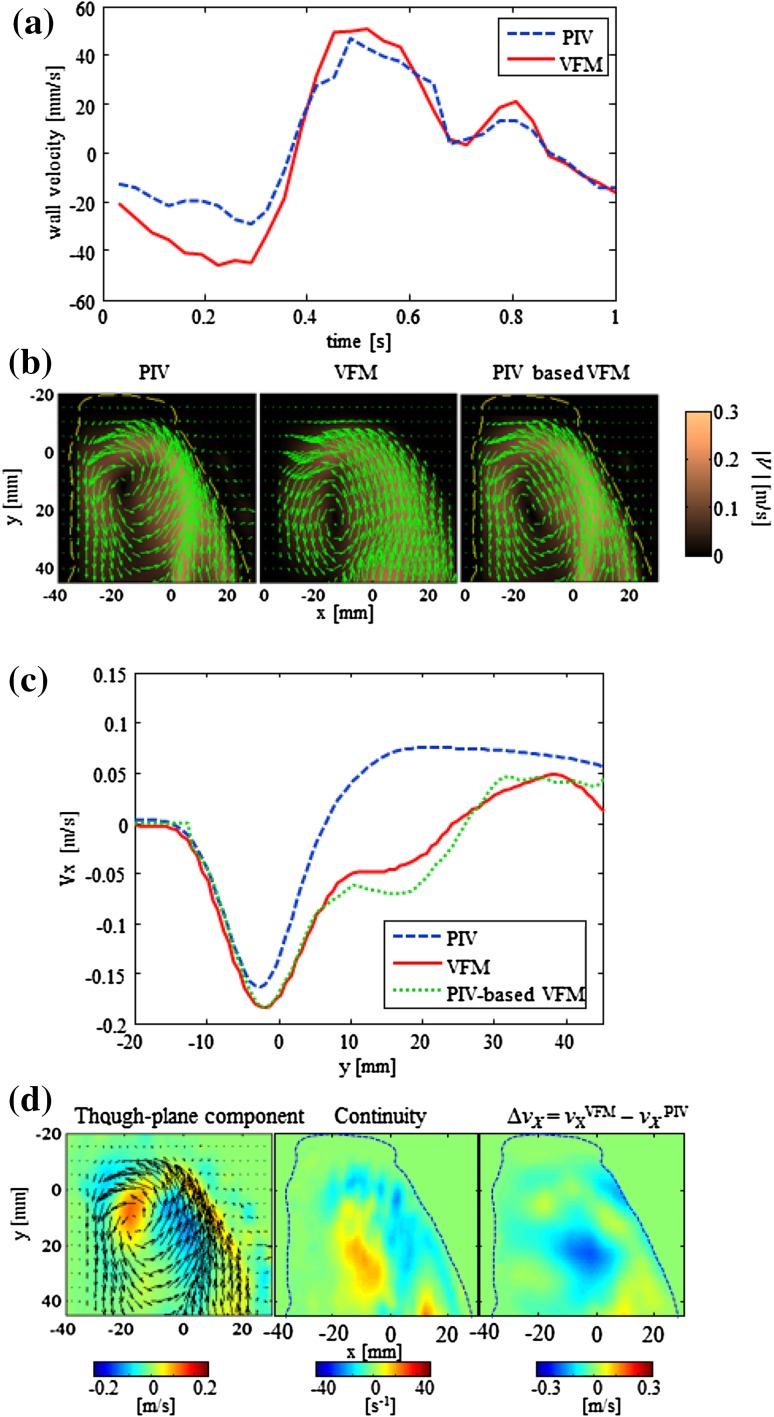
Comparison of wall velocities. **a** Horizontal component *v*_*x*_ of velocity of right wall at *y* = 15 mm, where wall motion is largest, **b** 2D vector mapping by PIV and VFM, and PIV reconstruction results (PIV-based VFM) at *t* = 0.9 s, **c** *v*_θ_ distribution along *y*-axis at *x* = 10 mm, and **d** intensity mapping of through-plane component of PIV vectors with overlay of 2D vectors (*left*), continuity equation (*middle*), and Δ*v*_x_ = *v*_x_^VFM^ *−* *v*_x_^PIV^ (*right*). *Dashed lines* indicate wall boundaries

The wall velocities detected by ultrasound tissue tracking were used as the boundary conditions for the VFM calculations. Tissue-tracking wall velocity has been compared with the wall velocity detected by PIV in Fig. 5a. The overall speed detected by echo tissue tracking is lower than that detected by PIV, resulting in a difference of 22 mm/s at most at *t* = 0.2 s, which is approximately 10% of the maximum flow speed. However, when VFM is recalculated using wall velocities detected by PIV (Fig. 5b), the flow dynamics appear to be identical to that in the original VFM. The RMS error was 8.4 mm/s at this place.

The VFM algorithm was applied to the PIV data to form PIV-based VFM data to enable the influence of the planar-flow assumption on error to be evaluated. The *v*_*x*_ of PIV-based VFM data was estimated from the wall velocity calculated from PIV data (*v*_*y*_) by solving the mass-conservation equation (Fig. 5b). PIV-based VFM data more closely resembles VFM data than PIV data in terms of the location of the vortex center. Distributions of *v*_*x*_ along the *x* = 10 mm line are plotted in Fig. 5c. The zero cross points of the original PIV data are at *y* = 7 mm and those of the VFM data are at 24 mm, whereas the zero cross points of the PIV-based VFM are at *y* = 26 mm. The maximum negative velocity is overestimated for both the VFM data and the PIV-based VFM data compared to that of the original PIV data. Another significant difference in the VFM and the PIV-based VFM from the original PIV data is that the slope of the velocity decreases at around *y* = 10 mm.

The through-plane velocity mapping obtained by stereo-PIV (left of Fig. 5d) indicates the presence of a three-dimensional flow around the center of the vortex. Significant deviation of *G* from zero is observed below the center of the vortex around *y* = 20 mm and *x* = 0 mm, indicating that the planer flow assumption was violated. The relevance of this violation to error in VFM velocity, Δ*v*_*x*_, is further investigated in Fig. 5d (right). Δ*v*_*x*_ is the discrepancy between VFM and PIV. Low Δ*v*_*x*_ can be observed around *y* = 20 mm and *x* = 0 mm.

## Discussion

The stereo-PIV results confirmed that there was a 30–40% through-plane flow of the total flow in the field of measurement, which suggests that the LV phantom is a reasonable platform for the 3D validation of VFM. There were several limitations in our phantom study. First, the placement of the mitral valve and aortic valve was on the same plane directly facing the apex of the phantom, thus failing to create the vortex transition that started at the mitral valve, traversed the chamber to the septum, and moved along the septum to the apex [1]. These mechanical valves were bileafelet, and therefore produced stenotic flow composed of separate jets which differs from that produced by natural valves [13]. Second, other dimensionless groups such as the Womersley number and Strouhal number did not have dimensional similarities whereas the dynamical similarity of the flow field within the phantom was ensured by approximately matching the Reynold’s number for the velocity validation purpose. For the mechanism validation such as vortex formation mechanism, other parameters such as Womersley number and Strouhal number would be important. For the current case, Womersley number and Strouhal number are 1.6 and 1.25 times the human heart situation, and they could be considered as the same order with the human heart situation. Nonetheless, the phantom simulated the nature of the 3D flow reasonably well and was in good agreement with the in vivo data acquired from previous studies [14].

The VFM data agreed well with the PIV measurements in all phases of the cardiac cycle. The average standard deviation of the velocity discrepancy was 4.5% over the CFM range (Fig. 4). The principle flow features and time-course transition of the main flow also agreed well (Figs. 3, 4).

The spatial resolution of the CFM signal was likely responsible for VFM underestimating the higher velocities, particularly during the rapid inflow (Fig. 4c). The fastest flow was observed in the transmitral jets, which had a narrower flow. These transmitral jets were mostly influenced by the azimuthal spatial resolution of the CFM signal. The radial resolution of the CFM signal was estimated to be a few millimeters at the depth of the mitral valve, which was considerably higher than that of PIV (0.4 mm). The difference between PIV data and VFM data is also apparent in Fig. 3 during the rapid inflow phase, where the two peaks are clearly separated in the PIV results but blurred into one large peak in the VFM results, resulting in high values in the discrepancy vector map. The figure also indicates that the lower spatial resolution of the CFM signal mostly affected rapid inflow. However, it should be noted that the two narrow and separated transmitral jets are unique to bileaflet mechanical valves, and the influence of spatial resolution in a healthy individual with normal valves is likely to be less.

Temporal resolution also affects VFM accuracy. One frame of acoustic data at 30 Hz that is used to construct VFM takes roughly 33 ms to acquire. Flows move as much as 33 mm while a single frame is acquired during rapid inflow, where the maximum speed reaches 1 m/s. This lag is most likely responsible for the difference in LEC paths between VFM and PIVs observed in Fig. 4e. It is noticeable to mention the future indices based on VFM. Although there are limitations of 2D measurement, properties defined by 2D flow fields such as circulation and vorticity can be expected to be reasonably accurate as long as the measured velocities are reasonably correct by their definitions.

Nonetheless, the effect of resolution of the CFM signal on the principle of flow dynamics (such as vortex formation and the motion of formed vortex centers) is minimal, as long as vortices remain larger than the resolution. The average vortex diameter of healthy individuals is estimated to be 9–13 mm [15], which is greater than the spatial resolution employed in this study. It should be noted that these CFM resolutions can easily be improved in numerous ways. For example, recent advances in echocardiography such as synthetic aperture imaging will doubtlessly improve the quality of VFM in this respect.

Differences in the locations of vortex centers along the *y*-axis are notable at *t* = 0.9 s in Fig. 3, indicating errors in VFM estimates, whereas the gross flow-pattern of VFM data is in excellent agreement with that of PIV data, as seen in Fig. 5. PIV-based VFM suggests that this error is neither caused by CFM nor tissue tracking, but by the 2D-flow assumption of the algorithm itself. The fact the error is caused by the existence of 3D flow is revealed in Fig. 5d. The location where greatest error Δ*v*_x_ occurs is similar to the locations where the 3D flow exists, which is indicated by the non-zero continuity value. Quantitatively speaking, this difference between PIV and VFM *v*_x_ components is relatively small (0.05 m/s) compared to the average flow speed. However, in terms of accurately analyzing flow patterns, a small difference is critical, and the violation of the planar-flow assumption suggests it plays an important role in determining the flow. It should be noted that the difference in the vortex-center location in an actual heart is estimated to be smaller by a factor of 1.6 by considering the dynamic similarity of fluids with almost the same Reynolds number.

Figure 5 suggests that the boundary conditions acquired by tissue tracking were reasonably accurate in comparison with the PIV data and did not contribute much to VFM calculation error. It should be noted that the edge of the phantom is possibly more clearly visible in echography than the actual cardiac wall and may have worked in favor of the tracking accuracy.

Error in VFM data was therefore mainly caused by CFM resolution (i.e., spatial and temporal) and the 2D-flow assumption. Whereas the former can be improved in many ways, the latter is likely to remain a problem due to the nature of the algorithm. Even though this study obtained accurate results when the through-plane flow component was 30%, it is obvious that patients’ cardiac hemodynamics differs, and the field of view changes with every operator. This is the most important limitation of VFM. While this study established a grounds for VFM accuracy in the case of cardio-hemodynamics of a healthy adult with an idealistic view, there will always be an uncertainty as to how much through plane flow occurs and how much error it causes. For example, when an abnormality in the LV wall motion is present, which is a likely case, the amount of through-plane flow may increase and so may the error it causes. Accordingly, to establish reliable clinical result, estimation of the uncertaintly caused by the through-plane flow [16] should be estimated in conjunction with the use of VFM. The reliability of VFM also varies according to the phase of cardiac cycle due to through-plain flow. Obviously, the algorithm works best in the case of the view with the least amount of through-plain flow.

On a final note, while VFM can also be applied to flow other than in LV, the nature of the VFM algorithm limits applicability of VFM primarily to the flow condition with wall boundaries. Also, because of error caused by the 2D-flow assumption, VFM is less appropriate for a short-axis view compared with long-axis apical, subcostal, and parasternal views.

## Conclusion

Accuracy and limitations of VFM estimation were investigated in reference to stereo-PIV data in vitro. An LV phantom was used for the validation that simulated the cardiac hemodynamics of a healthy adult, whose through-plane flow velocity was about 30% of the total flow velocity. The velocity field estimated by VFM agreed well with PIV measurements (in all phases of the cardiac cycle) with a correlation coefficient of 0.87 (*p* < 0.001). The transition paths of the center of the vortices shown with the local extremum of the magnitude of circulation in both the PIV data and VFM estimates were similar. The average standard deviation of the velocity discrepancy was 4.5% over the CFM velocity range. The discrepancy was mainly caused by both CFM resolution and the violation of 2D flow assumption. While VFM provided fairly accurate flow estimation in this phantom study, potentially greater error may occur in the case that LV flow contains more complex, 3D characteristics. t. Clinical evidence will be gathered in the future to justify the results obtained in this study.

## Appendix 1: derivation of vector flow mapping

The derivation of VFM has been detailed in previous reports [7, 8, 16]. Briefly, VFM assumes a 2D flow and calculates velocities by successively integrating the mass-conservation equation laterally with the boundary-wall velocity acquired by tissue tracking.

The ultrasound sector probe in the schematic in Fig. 1 scans the cylindrical coordinates, where *r* denotes the radial component and *θ* denotes the azimuthal component. Hence, the continuity equation is expressed as:

5 $$ r\partial_{r} v_{r} + v_{r} + \partial_{\theta } v_{\theta } + r\partial_{z} v_{z} = 0 $$

where *v* represents velocity, and subscripts *r*, *θ*, and *z* represent radial, azimuthal, and through-plane directions. If a planar flow is assumed, *v*_*z*_ is equal to zero. Given that boundaries exist at both ends of the radial direction, (5) can be integrated with respect to *v*_*θ*_ and rewritten as:

6 $$ v_{{_{\theta } }}^{\text{ccw}} \left( {r,\theta } \right) = v_{\theta ,a} (r) + \int_{{\theta_{a} }}^{\theta } {\partial_{\theta } v_{\theta } d\theta } $$

and

7 $$ v_{{_{\theta } }}^{\text{cw}} \left( {r,\theta } \right) = v_{\theta ,b} (r) + \int_{\theta b}^{\theta } {\partial_{\theta } v_{\theta } d\theta } $$

where the inside of the integral in this equation consists of the radial velocity, *v*_*r*_, that can be obtained by using color-Doppler velocities, the subscripts *a* and *b* denote the boundary conditions on the right and left of the cardiac wall, and the superscripts cw and ccw denote the calculation pathways. The existence of two boundary conditions allows two calculation pathways, as seen in Fig. 1. Because a no-slip condition is assumed, the boundary conditions are equivalent to the cardiac wall velocities measured by tissue tracking.

Equations (6) and (7) provide redundant information on azimuthal velocities. An error reduction scheme using a linear weight function is applied to improve accuracy [7] as:

8 $$ \begin{aligned} v_{\theta }^{{}} (r,\theta ) &= Wv_{\theta }^{\text{ccw}} (r,\theta ) + (1 - W)v_{\theta }^{\text{cw}} (r,\theta ) \hfill \\ W &= \frac{{\theta - \theta_{a} }}{{\theta_{b} - \theta_{a} }}. \hfill \\ \end{aligned} $$

## Appendix 2: correction of speed on sound

The resultant VFM velocity, *v* ^eqp^, calculated by ultrasound equipment was modified by simply multiplying the measured vectors by a correction factor, *C*_*f*_, as shown in Eq. (1). For a particular beam direction, the ultrasound equipment detects the Doppler-shift frequency, Δ*f*, and calculates flow velocity using the carrier frequency, *f*_*0*_, as

9 $$ v_{r}^{\text{eqp}} = \frac{\Delta f}{{f_{0} }}\frac{{c_{b} }}{2}, $$

although the real velocity, *v*_*r*_, is calculated as

10 $$ v_{r}^{{}} = \frac{\Delta f}{{f_{0} }}\frac{{c_{P} }}{2} $$

The relationship between *v*_*r*_^eqp^ and *v*_*r*_ is thus simply derived as

11 $$ v_{r}^{{}} = v_{r}^{\text{eqp}} C_{f} $$

For the azimuthal direction, the VFM velocity, *v*_*θ*_^eqp^, is calculated by the equipment on the basis of Eq. (12). Each term in the equation is examined to derive the correction for the speed of sound.

12 $$ v_{\theta }^{\text{eap}} (r,\theta ) = v_{\theta ,st}^{\text{eap}} (r) + \int_{{\theta_{st} }}^{\theta } {\partial_{\theta } v_{\theta }^{\text{eap}} } d\theta $$

The first term represents the wall velocity, *v*_*θ*,*st*_^eap^, which is calculated by tissue-speckle tracking using B-mode images. Note that the scale of entire B-mode images is simply shrunk by *C*_*f*_, because the set speed of sound is lower than the real speed or that in PEG400 as follows:

13 $$ r = r_{{}}^{\text{eqp}} C_{f} $$

Thus, the corrected wall velocity should be

14 $$ v_{{\theta ,{\text{st}}}}^{{}} = v_{{\theta ,{\text{st}}}}^{\text{eap}} C_{f} $$

For the second term in Eq. (12), the inside of the integration part is discretized and simplified using Eqs. (11) and (13) as

15 $$ \partial_{\theta } v_{{\theta ,{\text{st}}}}^{\text{eqp}} \cong - r^{\text{eqp}} \frac{{\Delta v_{r}^{\text{eqp}} }}{{\Delta r_{{}}^{\text{eqp}} }} - v_{r}^{\text{eqp}} \cong \frac{{\partial_{\theta } v_{\theta }^{{}} }}{{C_{f} }} $$

By substituting Eqs. (14) and (15) into Eq. (12), it is possible to write the correction as

16 $$ v_{r}^{{}} = v_{r}^{eqp} C_{f} $$

The correction using Eq. (1) is thus justified by Eqs. (11) and (16).
